# Executive Dysfunctions in Schizophrenia: A Critical Review of Traditional, Ecological, and Virtual Reality Assessments

**DOI:** 10.3390/jcm10132782

**Published:** 2021-06-24

**Authors:** Ernest Tyburski, Monika Mak, Andrzej Sokołowski, Anna Starkowska, Ewa Karabanowicz, Magdalena Kerestey, Zofia Lebiecka, Joanna Preś, Leszek Sagan, Jerzy Samochowiec, Ashok S. Jansari

**Affiliations:** 1 Institute of Psychology, SWPS University of Social Sciences and Humanities, 61-719 Poznań, Poland; 2 Department of Health Psychology, Pomeranian Medical University in Szczecin, 71-457 Szczecin, Poland; monika.mak@gmail.com (M.M.); zofia.lebiecka@gmail.com (Z.L.); joanna.pres@gmail.com (J.P.); 3 Memory and Aging Center, Department of Neurology, UCSF Weill Institute for Neurosciences, University of California, 675 Nelson Rising Lane, Suite 190, San Francisco, CA 94143, USA; andrzej.sokolowski@ucsf.edu; 4 Faculty of Psychology in Wrocław, SWPS University of Social Sciences and Humanities, 53-238 Wrocław, Poland; a.starkowska.pum@gmail.com; 5 Institute of Psychology, University of Szczecin, 71-017 Szczecin, Poland; ewa.karabanowicz@gmail.com (E.K.); magdalena@kerestey.net (M.K.); 6 Department of Neurosurgery, Pomeranian Medical University in Szczecin, 71-252 Szczecin, Poland; leszekm.sagan@gmail.com; 7 Department of Psychiatry, Pomeranian Medical University in Szczecin, 71-457 Szczecin, Poland; samoj@pum.edu.pl; 8 Department of Psychology, Goldsmiths, University of London, New Cross, London SE14 6NW, UK; a.jansari@gold.ac.uk

**Keywords:** virtual reality, schizophrenia, executive functions, cognitive functions, ecological validity, neuropsychology, psychopathological dimensions

## Abstract

In recent years, interest has grown in measuring executive function in schizophrenia with ecological and virtual reality (VR) tools. However, there is a lack of critical analysis comparing those tools with traditional ones. This paper aims to characterize executive dysfunction in schizophrenia by comparing ecological and virtual reality assessments with traditional tools, and to describe the neurobiological and psychopathological correlates. The analysis revealed that ecological and VR tests have higher levels of verisimilitude and similar levels of veridicality compared to traditional tools. Both negative symptoms and disorganization correlate significantly with executive dysfunction as measured by traditional tools, but their relationships with measures based on ecological and VR methods are still unclear. Although there is much research on brain correlates of executive impairments in schizophrenia with traditional tools, it is uncertain if these results will be confirmed with the use of ecological and VR tools. In the diagnosis of executive dysfunction, it is important to use a variety of neuropsychological methods—especially those with confirmed ecological validity—to properly recognize the underlying characteristics of the observed deficits and to implement effective forms of therapy.

## 1. Introduction

Executive dysfunction, cognitive deficits, positive and negative symptoms, and disorganization are key psychopathological symptoms in schizophrenia; they are consequences of abnormalities in the structural and functional levels in the brain [1,2]. The cognitive deficits affect attention, verbal and episodic memory, and visuo-spatial processes [1,2]. It is worth noting that it has been claimed that executive dysfunction constitutes the most specific set of neuropsychological symptoms in schizophrenia [3,4,5]. Although executive dysfunction is not included in the diagnostic criteria for schizophrenia in the Diagnostic and Statistical Manual of Mental Disorders-5th Edition (DSM-5) [6], the international group of experts who authored the manual define them as separate domains of cognitive impairments and indicate that they are important aspects of schizophrenia. Investigating the nature of executive dysfunction is very important, especially due to the fact that they affect as many as 1% of the general population, regardless of country or cultural background, causing significant impairment in the social, professional, and family life of patients and leading to profound disability [7,8,9].

Currently, there is ongoing debate as to whether distinct types of schizophrenia can be linked with specific executive disorders [10,11]. Numerous authors point out the need to analyze particular subtypes of the disorder separately (e.g., those with predominantly positive or negative symptoms [12]). Due to the heterogeneity of schizophrenia symptoms, it seems valid to distinguish more homogeneous groups of patients within this one condition (such as negative and positive syndromes). Carpenter, Heinrichs, and Wagman [13] propose the category of Deficit Schizophrenia (DS)—a subtype of the disorder with predominantly primary negative symptoms, which are stable over time. Negative symptoms include poverty of speech, social withdrawal, limited speech content, restricted affect, and apathy [14]. Numerous reports have demonstrated the validity of the diagnosis of DS, due to, among other things, variations in genotype distribution [15], functional and structural differences in the brain [16], and cognitive and executive function differences [17,18]. However, research on the occurrence of executive dysfunction in DS has yielded inconsistent results. Some studies suggest that, compared to non-deficit patients (NDS; patients without deficit psychopathological symptoms), those with DS manifest difficulties with regards to cognitive inhibition, problem-solving, and verbal and non-verbal cognitive flexibility [19,20]; however, other studies have failed to confirm these results (for a detailed overview see: [21]).

One of the most important challenges in contemporary neuropsychology is the use of innovative technological methods to diagnose and treat schizophrenia patients [22]. Moreover, it is of utmost importance to develop tools that contribute to understanding and predicting patients’ behavior in real-life situations [23]. There are several systematic reviews [24,25], several selective reviews, and one meta-analysis [26] about assessment and treatment using VR in different clinical populations. Other papers explore VR in the schizophrenia spectrum in the broad context of different cognitive functions [27,28,29]. However, none of these seem to offer an extensive analysis of executive dysfunction measured by VR in schizophrenia. To our knowledge, no previous papers detail the complex characteristics of and compare traditional, ecological, and VR tools. Despite the well-established links between executive impairments measured by traditional tools and psychopathological dimensions e.g., [30], links between measurement by ecological and VR tools and the aforementioned dimensions have been severely neglected in systematic reviews. This is all the more surprising as knowing these relationships helps us understand the nature of schizophrenia and to organize interdisciplinary diagnostic and support systems for patients [31]. Moreover, systematic reviews lack information about the relationships of white matter integrity or functional changes in the brain with executive functions measured by ecological and VR tools. Successfully finding biological markers would enable faster diagnosis, earlier implementation of effective treatment, and improved quality of life [32]. Given these limitations and the paucity of comprehensive analyses in the available review papers, we formulated the following objectives. The primary aim of this review was to characterize executive dysfunction in schizophrenia in conditions resembling everyday situations by comparing traditional, ecological, and VR tools in terms of the ecological dimensions of verisimilitude and veridicality. Our secondary goal was to describe the neurobiological and psychopathological correlates of that dysfunction.

## 2. Nature of Executive Functions

Executive functions constitute an important subject of interest to researchers in various scientific disciplines. These processes are an important construct in modern neuropsychology and they may be defined differently depending on the adopted theoretical approach [33]. It is commonly accepted, however, that they mediate between cognition (information processing systems) and action (i.e., the performance of activities) [34]. The theoretical background of executive functions can be traced back to Luria’s research [35]. He distinguished three functional systems in the human brain: (a) an arousal and attention unit (limbic and reticular activation system); (b) a unit that receives, stores, and analyzes information (posterior neocortex); and (c) a unit that plans, organizes, and regulates behavior and cognition (frontal lobes). In his approach, regulation is defined as the ability to change one’s actions during the execution of a particular task, which often takes place with the involvement of linguistic processes [36]. The capacity to contrast the outcomes of a given behavior with its initial purpose is defined as control. Finding a solution to a problem involves analyzing initial conditions, developing a specific plan (strategy), implementing the relevant operations, and comparing the result with the initial data [37].

On the other hand, Lezak, Howieson, and Loring [38] propose that executive functions form a comprehensive set of processes or mental abilities used in the execution of purposeful actions, enabling one to adapt to new situations. Lezak [38] clinically describes executive functions in terms of four main domains: volition, purposive action, effective performance, and planning. The first stage involves a conscious, future-oriented decision or intention to carry out a deliberate action. The next stage, planning, happens once the goal has been set: a sequence of steps necessary to solve the problem or achieve the goal is identified. Purposive action is the process of turning intentions and plans into a specific behavioral act; this requires the monitoring and correction of one’s course of action as well as the modulation of the tempo and intensity of one’s own reactions. Another important element of executive functions is working memory, introduced by Baddeley [39], which consists of a central executive, phonological loop, and visual-spatial sketchpad. Working memory is also considered an aspect of executive functions in the model proposed by Diamond [40].

Following these important formulations, researchers currently understand executive functions as the mental abilities responsible for, inter alia: (a) planning and organization, (b) anticipation and focus of attention, (c) initiation of activity, (d) self-monitoring and impulse control, (e) working memory, (f) mental flexibility and the ability to make use of feedback, and (g) choosing effective strategies for problem-solving [41]. These functions also constitute an executive process that designates mental resources and monitors, inhibits, and controls other mental processes and behavioral reactions, thereby reinforcing better adaptation to the external environment [42]. Depending on the dynamics of a situation, they facilitate the adjustment of behavior and tailoring of reactions [34]. Cognitive and behavioral control constitute two especially important executive domains [43,44]. Studies have shown a factorial structure of executive functions in healthy individuals by analyzing multiple measures of executive performance. For instance, Testa, Bennett, and Ponsford [45] performed a factor analysis of 19 neuropsychological tests on 200 healthy people and differentiated six largely independent factors: set-shifting and interference management, prospective working memory, task analysis, strategy generation and regulation, response inhibition, and self-monitoring and set-maintenance. However, Miyake et al. [46] distinguished only three central executive domains (shifting, inhibition, and updating). The authors suggested the relative independence of these functions but noted that they share certain common characteristics.

It is uncertain whether the same factor structure would be found using different ecological and VR tools, and more research on this topic is needed. Additionally, the factor structure of executive functions identified in healthy populations may differ from that of clinical populations (e.g., for schizophrenia patients, see: [19]). Despite different theoretical models of executive functions and repeated factor analyses, there are still inconsistencies in their definitions and number of components; hence, “executive functions” can be considered as an umbrella term indicating a “wide range of cognitive processes and behavioral competencies” [33].

## 3. Brain Correlates of Executive Dysfunction in Schizophrenia

To improve the diagnosis and treatment of executive dysfunction in schizophrenia, it is necessary to understand the complex relationship between executive functions and the brain’s structure and activity [47]. Generally, the main cause of executive dysfunction is believed to be abnormalities in different areas of the prefrontal cortex (e.g., dorsolateral, ventrolateral, and anterior cingulate cortices) and their connections with other brain regions, which are part of complex neural circuits [48]. On the basis of broad analysis of evidence from structural and functional neuroimaging studies, Orellana and Slachevsky [4] proposed dividing all neurocognitive theoretical models of schizophrenia into two categories. The first category includes neuroanatomical models that postulate that the executive impairments are caused by dysregulation of specific brain circuits and regions: disruption of the fronto-striato-thalamic system, disruption of the frontotemporal system, and disruption of the frontoparietal systems. The second category comprises cognitive models that postulate that specific cognitive disorders are responsible for the symptomatology of schizophrenia, such as Cohen’s model of updating internal representations of contextual information and Frith’s model of disorder of consciousness or self-awareness that impairs the ability to think with meta-representations.

One proposition that goes beyond the traditional approach is the functional neuroscientific paradigm in which links are drawn between particular brain regions and specific functions. Recently, researchers have identified six brain neural networks involved in cognitive functions, but according to the new paradigm, executive functions are performed based on interactions between three large-scale brain networks, which do not have strictly defined, localized frameworks [49,50]. This approach highlights the role of whole-brain functional networks engaged in cognitive processes. These networks have been demonstrated to include local neurons (located in areas of particular importance for specific mental processes) that interact in synchrony with each other and at the same time stimulate entire distal cell populations (in areas that are often remote from one another) [51]. The central executive network (CEN), consisting primarily of the dorsolateral prefrontal cortex (DLPFC) and posterior parietal cortex, is activated in healthy subjects when performing tasks involving executive functions associated with action-orientation and with a primary focus towards the outside world [52]. In turn, the default mode network (DMN), encompassing the ventromedial prefrontal cortex, posterior cingulate cortex, and precuneus, is deactivated during the performance of tasks that are associated with focus on oneself [49,50]. Finally, the salience network (SN), involving activations in the anterior insula and the dorsal anterior cingulate cortex, deals with the dynamic shifting between the activity of the CEN and DMN networks, thus redirecting attention resources onto the most important task at any particular moment [53]. One recent meta-analysis [54] of fMRI research in schizophrenia has confirmed changes in the activity of structures involved in these large-scale functional networks.

The disconnection hypothesis is a model of the etiopathogenesis of schizophrenia that links it to the disturbance of the aforementioned neural networks [55]. Such impaired communication is the result of, among other things, structural alterations during ontogenetic development of the structures that constitute these functional networks [56,57]. Although studies using functional neuroimaging have repeatedly suggested abnormalities in the network of communication between the CEN, DMN, and SN in schizophrenia patients, not much is currently known about the structural connections within these networks [58]. Executive disorders due to abnormalities in the structure and activity of the prefrontal and thalamic networks are seen as the most characteristic neuropsychological symptoms of schizophrenia [4,59]. Drawing on 41 functional magnetic resonance imaging studies, Minzenberg et al. [60] demonstrated that executive deficits in schizophrenia are accompanied by decreased activity in the left DLPFC, rostral and dorsal ACC, left thalamus, and inferior/posterior cortical areas. Some reports indicate that, compared to healthy controls, patients with schizophrenia have structural abnormalities within the CEN, including reduced volume in the lateral PFC as well as in posterior areas of the parietal lobes [61] and reduced integrity of the nerve fibers connecting these areas [62]. Other reports show that structural abnormalities are present also in the cortical areas [63] and connections [64] of the DMN. Finally, there are reports of structural abnormalities in the anterior cingulate and insula [65] and the integrity of nerve fibers connecting the brain areas [66] that constitute the SN.

Reduced volume of the DLPFC and the ACC as well as reduced integrity of the cingulum bundles (CB) in schizophrenia patients is linked to impaired ability to generate rules and problems with abstract thinking [67,68,69,70,71]. Furthermore, reduced integrity of the frontal portion of the CB [64,72] and the anterior limb of the internal capsule (ALIC) [62] has been found to be linked to deficits in cognitive inhibition of dominant verbal responses. Other reports have also shown that reduced integrity of the ALIC [73] and fornix [74] correlate with impaired cognitive flexibility. However, some studies did not find a relationship in patients with schizophrenia between executive functions and white matter integrity in the superior longitudinal fasciculus, corpus callosum, anterior cingulum, and forceps minor [75,76].

## 4. Old and New Paradigms for Assessing Executive Functions in Schizophrenia

Different paradigms for assessing executive functions have been discussed in the field of neurocognitive psychology. Burgess et al. [77] describe three elements on which neuropsychological assessment can focus: constructs, operations, and functions. Constructs denote hypothetical cognitive resources whose existence is inferred from research findings (e.g., cognitive inhibition). Operations are the individual component steps of cognition that are not directly observable but can be inferred from a combination of task analysis and changes in some dependent variable (i.e., an observable correlate of cognitive inhibition, such as reaction time on the Stroop task). Functions, in contrast, are directly observable behavioral output—the product of a series of operations. They are usually understood in terms of a goal, instruction, or intention to act, and therefore it is usually clear what constitutes success or failure, such as naming the color of words on the Stroop task. The old paradigm, which aims to diagnose brain pathology, is the basis of most traditional tools. Due to low process-behavior correspondence and non-material-specific processing, the field of executive functions is especially heavily construct-oriented. The ecological paradigm is, on the other hand, oriented toward functions, which is of great clinical importance for diagnosing patients’ everyday cognitive abilities and disabilities (i.e., at school or work). Research on the construct validity of ecological, function-oriented tools has revealed correlations between traditional, construct-oriented tools and ecological ones, but these associations turn out to be lower than ideal for establishing construct validity [78,79]. Despite the fact that tools developed in both paradigms measure executive functions, they are understood in slightly different ways, which raises the question of whether they actually measure the same construct.

The ecological paradigm highlights the importance of ecological validity in neuropsychological testing, which is understood as “the degree to which test performance corresponds to real-world performance” (p. 192, [80]). There is a need in neuropsychology to develop research on the ecological validity of assessment and rehabilitation in the context of clinical and forensic applications [81,82]. There are two prominent approaches to defining crucial aspects of this type of validity. On the basis of the work of Kvavilashvili and Ellis [83], Burgess et al. proposed the concepts of the representativeness of a task, understood as “the extent to which a clinical test corresponds in form and context to a situation encountered outside the laboratory” (p. 195, [77]), and the generalizability of test results, understood as “the degree to which poor performance on the test will be predictive of problems outside the laboratory” (p. 195, [77]). Franzen and Wilhelm [84] defined ecological validity in terms of verisimilitude and veridicality. The concept of verisimilitude refers to “the degree to which the cognitive demands of a test theoretically resemble the cognitive demands in the everyday environment” (p. 182, [84]). Veridicality is defined as “the degree to which existing tests are empirically related to measures of everyday functioning” (p. 182, [84]). While veridicality can be measured by correlation with other measures of functional outcome, including naturalistic observations, clinical scales, and questionnaires, verisimilitude lacks empirical indicators, and a given tool can be considered valid if it was constructed with verisimilitude in mind.

**Figure 1:**
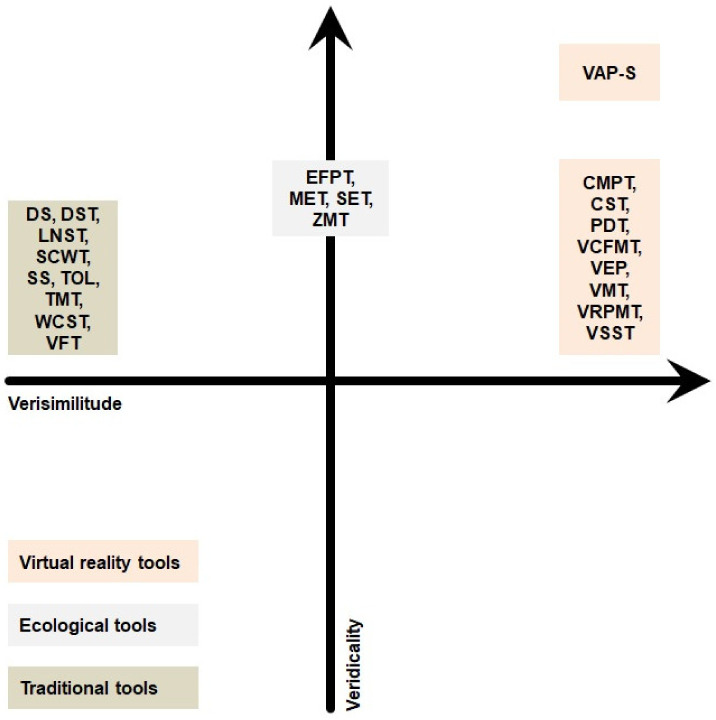
Tools for assessing executive functions in terms of their ecological validity level (verisimilitude and veridicality). Verisimilitude as a continuous dimension: stimuli and activities without features of real-life—stimuli and activities with features of real-life. Veridicality as a continuous dimension: lack of correlation of functional outcome and behavioral observation—correlation of functional outcome and behavioral observation. CMPT = Computerized Meeting Preparation Task; CST = Computerized Shopping Task; DS = Digit Span; DST = Digit Sequencing Test; EFPT = Executive Function Performance Test; LNST = Letter Number Sequencing Test; MET = Multiple Errands Test; PDT = Plan-a-Day Test; SCWT = Stroop Color Word Test; SET = Six Elements Test; SS = Spatial Span; TMT = Trail Making Test; TOL = Tower of London; VAP-S = Virtual Action Plan—Supermarket; VCFMT = Virtual Cognitive Flexibility Measurement Task; VEP = Virtual Egyptian Pyramids; VFT = Verbal Fluency Test; VMT = Virtual Maze Task; VRPMT = Virtual Reality Prospective Memory Test; VSST = Virtual Supermarket Shopping Task; WCST = Wisconsin Card Sorting Test; ZMT = Zoo Map Test.

In this paper, we adopt the approach of Chaytor & Schmitter-Edgecombe [80] in describing and classifying neurocognitive tools for measuring executive functions in schizophrenia as it is more research-focused, whereas the approach of Burgess seems more clinically oriented. We divided diagnostic tools into three basic categories: (a) traditional; (b) ecological; and (c) virtual reality (VR) methods, which can be understood as a subclass of ecological tools, but due to their graphic realism and the actual activities the participant undertakes in the environment, resulting in higher verisimilitude, we decided to present them as a separate category. We chose ecological and VR tools on the basis of their innovative character and clinical value in schizophrenia diagnosis; for traditional tools, we selected the most used and researched ones. Figure 1 shows the tools used to assess executive functions in schizophrenia in terms of the dimensions of verisimilitude and veridicality. Complementary Table 1 presents more detailed characteristics of verisimilitude, evidence for verisimilitude, and diagnostic validity.

## 5. Assessment with Traditional Tools

Traditional pencil and paper tests are designed to measure executive functions in new or non-standard situations. Regardless of the suggested theoretical approach, research based on the use of standard methods indicates that patients with schizophrenia exhibit disturbances in inhibition, monitoring, attention shifting, rule generation, abstract thinking, planning, and working memory; these disturbances hamper the execution of goal-oriented tasks (see: Table 2) [130,131]. Furthermore, due to the fact that executive symptoms in patients with schizophrenia are often dissociated, meaning that the processes are subject to differential impairment, patients are often diagnosed with “dysexecutive syndrome”, a term often used to describe these different symptoms [132]. Such a diagnosis is based on patients’ poor performance on traditional pencil and paper tests, such as the Stroop Color Word Test (SCWT) [1,87,88], the Tower of London test (TOL) [93,94,95], the Trail Making Test (TMT) [1,87,88], the Wisconsin Card Sorting Test (WCST) [1,2,87,88], the Verbal Fluency Test (VFT) [1,87,88], the Digit Span test (DS) [1,87,99,100], the Digit Sequencing Test (DST) [10], the Letter Number Sequencing Test (LNST) [1], and the Spatial Span test (SS) [99,100]. The traditional neuropsychological tools used to assess executive functions in schizophrenia are shown in Table 3.

Unfortunately, these tools typically have a similar structure and methodology to the first methods used in the early twentieth century, (e.g., the Stanford-Binet Intelligence Scale) and do not reflect patients’ difficulties in real life; in effect, these tests are characterized by low levels of veridicality, confirmed by correlations on the level of 0.20–0.30 with functional outcome scales (see: Table 1 and Figure 1) [85]. Additionally, these tools lack verisimilitude, as they were not originally designed for their current purpose—to assess executive functions in patients with psychiatric disorders—because they were either constructed as experimental investigations of basic cognitive systems in healthy persons (e.g., WCST) or for testing frontal lobe functions in patients with anterior lesions (e.g., TOL). Therefore, it might not be appropriate to draw conclusions concerning different aspects of executive dysfunction in schizophrenia based on performance on such tests. Besides, since traditional tools neglect the influence of emotional and social processes by diverging from the real-life situations with which patients may have problems, it is impossible to assess the practical effects of executive functions. Another limitation is that during standard psychological testing, patients usually perform only one task, while, in real life, situations usually require multitasking—carrying out a number of tasks simultaneously, which often compete with each other at the same time [110,133]. Unlike in the laboratory, real-life problems are mostly open-ended in nature, which is not reflected in the fixed goals and defined structure of traditional tools [107]. Many patients with schizophrenia who achieve satisfactory results on traditional tests can be impulsive, easily distracted, and have problems with using feedback, which may be reflected in problems with real-life situations. These limitations can be more adequately captured by more ecological tools [113]. Despite these limitations, traditional tools are often used because of: familiarity, long tradition, availability, price, short assessment time, and satisfactory diagnostic validity (see: Table 1).

## 6. Assessment with Ecological Tools

Ecological methods are based on a new methodology that attempts to measure executive functions as they are used in real life situations. These methods are characterized by a high level of representativeness of function, which means that they resemble situations occurring outside the research laboratory, and verisimilitude, which indicates how well they mirror the cognitive demands of authentic real-life challenges [80]. Patients with schizophrenia have demonstrated limitations in their ability to solve open-ended problems, difficulties performing simultaneous tasks, as well as deficits in planning, abstract thinking, and prospective memory (which, together, are necessary for completing operations in conditions resembling true-to-life situations) in their poor performance on ecological tests such as the Executive Function Performance Test (EFPT) [104], the Multiple Errands Test (MET) [106,108], the Modified Six Elements Task (MSET) [110], and the Zoo Map Test (ZMT) [110]. Descriptions of these ecological neuropsychological tools used to assess executive functions in schizophrenia are given in Table 4.

Ecological tools have particularly good verisimilitude and veridicality (see: Table 1 and Figure 1); that is, performance in the task situation is predictive of performance in situations outside it and the context and form of these tools corresponds to situations encountered in real life—an important factor for clinical diagnosis. Ecological tools are more useful for identifying executive dysfunction in patients with schizophrenia due to the fact they measure executive skills in a more integrated way, through tasks that are closer to real-life situations, instead of separated theoretical components (e.g., planning or shifting) [107].

## 7. Assessment with Virtual Reality

VR is an advanced interactive computer technology that generates a three-dimensional environment [26]. It is characterized by a high level of immersion. Immersion is a multidimensional construct constituted by the objective characteristics of the virtual system, perceptual responses to the virtual system, subjective responses to the narrative content, and subjective responses to challenges in the virtual environment [134,135]. One’s subjective immersion in a virtual environment is a crucial feature of VR and is characterized by two factors: place illusion, defined as subjective sense of presence in a virtual environment, and plausibility illusion, namely the VR’s capacity to adequately react to the individual’s behavior [25]. VR tools are often more complex than the traditional tests used to measure executive functions. They are characterized by various non-linear goals, and thus are able to measure multiple processes. Relatively lengthy immersion in a realistic environment allows the accurate assessment of behavior in everyday situations [136].

There are three main types of VR assessments, which differ in terms of level of immersion. The least immersive is a simple system that uses displays on monitors [28]. Moderately engaging methods involve the projection of images on a wall while participants use shutter glasses to view the scene. The most immersive systems involve the use of head-mounted displays [25]. The most engaging environments are used in the therapy of various clinical groups, for example, schizophrenia and anxiety disorders. In scientific research, simple and less immersive systems are most often used since they enable the control of the basic characteristics necessary for strict experimental design [24].

Research based on VR methods has confirmed the presence of specific executive difficulties in patients with schizophrenia, hindering their performance of complex everyday activities, such as shopping, using public transport, or preparing a staff meeting in a business office [24,25,137]. Although, like traditional tools, some VR tests were not developed for patients with schizophrenia, evidence shows that they have good diagnostic validity (see: Table 1). Using VR tools, it has been shown that schizophrenia patients have impaired integration of visual and auditory stimuli. Moreover, Ku et al. [125] suggested that patients with schizophrenia have impaired multimodal integration and working memory integration based on asking them to react to and interpret multimodal stimuli that had to be memorized for a certain time period. Jeonghun Ku, Kim, and Kim [138] showed that patients with schizophrenia cannot properly systematize or integrate the integrated stimuli that occur in actual situations. A VR system was developed to administer multimodal stimuli such as integrated visual and auditory stimuli. Patients were assessed and compared in terms of their cognitive flexibility and working memory abilities by making them experience and react to multimodal stimuli. Sorkin, Weinshall, Modai, and Peled [139] examined sensory integration within schizophrenia patients’ working memory by means of a virtual maze. Participants navigated through a series of rooms, repeatedly having to choose from three doors. Each door had three characteristics (color, shape, and sound) and only one combination of characteristics would open the door. Participants had to discover the rule and implement it. When the task was over, each participant was given a performance profile which included response time, error scores, strategy, and navigation ability. Based on these profiles, a classification procedure was able to correctly identify 85% of the schizophrenic patients. Furthermore, the patients did not exhibit any exceptional repetition of responses regardless of the cessation of stimuli (traditionally known as “perseveration”), as often happens in such patients. This only occurred when the task was not properly explained to the participants.

**Figure 2:**
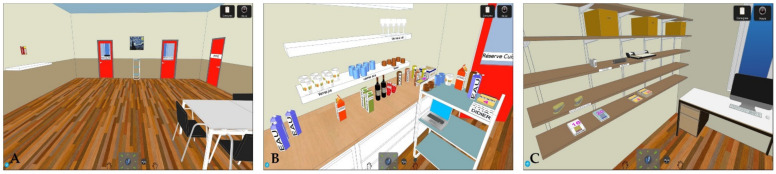
Sample pictures presenting the main room, person-avatar, and office material area from the Computerized Meeting Preparation Task (CMPT) [119]. Reprinted with permission from [119]. Copyright 2014 Elsevier. Participant‘s first person view: (**A**) in the main room where the meeting takes place, (**B**) in the kitchen, and (**C**) in the office material area.

**Figure 3:**
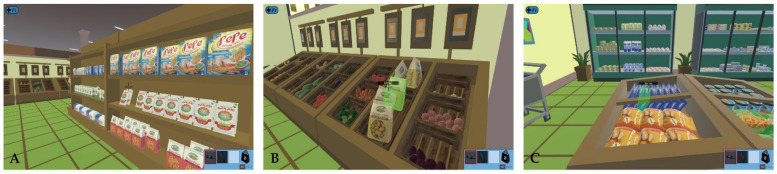
Sample pictures presenting the participant’s first-person view while collecting items on the Virtual Supermarket Shopping Task (VSST) [129]. The names of the products are visible when pointed at. Reprinted with permission from [129]. Copyright 2021 Czech Academy of Sciences. Participant‘s first person view: (**A**) while collecting an item from the shelf, (**B**) while collecting fruits and vegetables, and (**C**) while collecting products from the fridge.

Other specific executive difficulties in patients with schizophrenia have been demonstrated in their poor performance on, inter alia, the Virtual-Action Planning Supermarket (VAPS) [116], the Virtual Cognitive Flexibility Measurement Task (VCFMT) [118], the Computerized Meeting Preparation Task (CMPT; see: Figure 2) [119,120], Computerized Shopping Task (CST) [121], Plan-a-Day Test (PDT) [123,124], Virtual Egyptian Pyramids (VEP) [125], Virtual Maze Task (VMT) [126], Virtual Reality Prospective Memory Test (VRPMT) [128], and Virtual Supermarket Shopping Task (VSST; see: Figure 3) [129]. The VR neuropsychological tools used to assess executive functions in schizophrenia are shown in Table 5.

To summarize, VR methods involve measuring executive functions in virtual conditions. They enable the simulation of physical space in the laboratory environment that emulates the real world [27]. Moreover, such methods can allow participants to manipulate three-dimensional objects and even interact with avatars representing other people; they also give researchers the opportunity to make a more detailed analysis of the patient’s behavior during the performance of a task [26]. Another important advantage of tools based on this technology is the so-called sense of immersion, or the feeling of presence in the virtual world [134]. VR assessment of executive functions tends to be similar to those performed with other ecological methods in terms of, for example, multitasking, open problems, and similarities to real situations. Executive functions measured in a virtual environment (e.g., activities like shopping and managing finances) are linked to the processes that are assessed by ecological tools [116,121,123]. Due to the fact that virtual reality simulates natural environments and participants have a sense of immersion, it makes possible the diagnosis of impairments of the executive functions that are used in everyday activities. Although schizophrenia patients exhibit problems with cognitive and behavioral inhibition, so far, most VR tools measure only planning, decision-making, and multitasking. Future studies should investigate inhibition tasks in VR, using, for example, the Stroop paradigm [78,140,141].

In his recent work, Parsons [142] presented a list of potential risks of using VR in clinical conditions, including: (a) simulator sickness, (b) high fidelity stimulus presentations, experience intensification, and information overload, (c) depersonalization, derealization, and dysfunctional re-entry into the real, (d) virtual environments with vulnerable populations, and (e) therapeutic misconceptions. Additionally, VR tools tend to be costly, especially highly immersive methods (e.g., head-mounted displays). Patients have to learn how to use often complicated human-computer interfaces, which may be difficult for individuals with severe perception and memory impairments. For older patients, computer anxiety may also be part of the problem [143]. For the welfare of patients, it is essential that technology support clinicians in their therapeutic efforts, not substitute for them. The therapeutic relationship is exceedingly important for both diagnosis and neuropsychological interventions.

## 8. Relationship of Executive Dysfunction with Psychopathological Symptoms

Some studies have proposed that psychopathological symptoms could partially overlap with executive dysfunction in schizophrenia; for example, it has been found that negative symptoms correlate with decreased abstract reasoning and impaired set shifting [144], positive symptoms seem to only correlate with deficits in inhibition [145], and disorganization symptoms correlate with difficulties in abstract thinking and impairments in cognitive flexibility [146,147]. Some meta-analyses further confirmed the relationships of both negative symptoms and disorganization with executive impairment measured using traditional tests, with significant correlations being in the small-to-moderate range [30,148]. However, correlations for positive symptoms were nearly zero. The correlations between various tests of executive functions were significantly different for disorganization and negative symptoms. For example, there were stronger correlations between negative symptoms and the TMT, VFT, and SCWT than with the WCST and working memory tasks, while there were stronger correlations between disorganization symptoms and the TMT, SCWT, and WCST than with the VFT and working memory tasks. Patients who had stable clinical profiles exhibited significantly stronger correlations with executive impairment than did those with remitting or relapsing illnesses. Additionally, negative symptoms and disorganization both correlated with general intellectual function (current IQ).

According to Thai et al. [110], only two studies have looked into the relationship between psychopathological symptoms and executive dysfunction using ecological tests. There were no significant correlations of subtests from the Behavioral Assessment of the Dysexecutive Syndrome (BADS) battery with negative, positive, or disorganized symptoms [111,149]. In contrast, as assessed with Activities of Daily Living, negative symptoms were linked to omissions during the choice of menu and greater delays between the first and the last dishes, as well as the number of planning and repetition errors when cooking the meal [150].

Only a few studies simultaneously focus on the link between psychopathological symptoms and executive functions measured by VR tools in patients with schizophrenia. For example, on the Computerized Shopping Task, Larøi et al. [121] showed that positive, negative, and general symptoms were negatively correlated with the total times participants consulted the list and the number of correct articles. Moreover, Josman et al. [116] showed that negative symptoms correlated with all indices of the Virtual Action Plan—Supermarket (VAP-S): trajectory duration, distance covered, purchases, correct actions, number of stops, and total duration of stops. However, Laloyaux et al. [119] found no correlations between psychopathological symptoms and different aspects of executive function as measured by the Computerized Meeting Preparation Task (CMPT). A recent meta-analysis of the Positive and Negative Syndrome Scale suggests five main psychopathological symptoms in schizophrenia: negative symptoms, positive symptoms, disorganization, resistance, and affect [151]. However, to the best of knowledge, this approach lacks data on the relationship between psychopathological dimensions and results on VR tools that assess executive functions in patients with schizophrenia.

## 9. Challenges and Future Directions

Despite the growing body of literature on executive function assessment and rehabilitation in schizophrenia, some unanswered questions still remain. One of the questions concerns the relationship between executive deficits and structural abnormalities within cortical areas of the brain and in the connections that create the three large-scale functional networks. Most importantly, studies that have indicated such links have been conducted using traditional methods to assess executive functions. There is little data on a possible link between the structure and activity of the described neural networks and the executive functions engaged by daily activities in patients with schizophrenia. Such data can be collected only through ecological testing and VR methods. Future research should be based on paradigms that allow the use of VR assessment of executive functioning with methods of functional neuroimaging. This approach has been already used in research on memory, learning, language production and comprehension, and social interaction (with the use of functional brain neuroimaging, e.g., [126,152,153,154,155] and with event-related potentials based on electroencephalography, e.g., [156,157]). Gainsford et al. [158] made the interesting proposal of combining VR methods with non-invasive brain stimulation in the social cognition domain, which would also be interesting to use to investigate executive function in schizophrenia.

Technological advancements in the construction of tools have led to the emergence of new questions and challenges. The questions concerning VR posed by Freeman [25] about learning transfer from VR to the real world, the possibility of the application of complex psychological treatment techniques to VR, and its benefits for patients in real life are still valid. We consider the following research questions to currently be important in this field: (a) How can the verisimilitude of neurocognitive tools be empirically verified? (b) What are the relationships between the structure and activity of functional brain networks and executive functions in real-life situations?

The literature analysis described in this paper reveals important issues that should be considered in future research. One idea for measuring the verisimilitude of VR is to use a panel of experts containing, in addition to clinicians, individuals who actually work in a given environment (e.g., shop workers and regular customers in the case of supermarket environments). As self-description questionnaires and clinical ratings do not provide a full picture of a patient’s behavior outside the clinic, we suggest that veridicality should be assessed in a more precise way, preferably by naturalistic observation in appropriately chosen situations [117]. In addition to ecological validity, ecological tools, especially VR methods, should be investigated in terms of the classical psychometric measures of different types of reliability and validity, including diagnostic validity, in terms of sensitivity, specificity, positive predictive power, and negative predictive power. In assessing test-retest reliability for executive functions measures, it is important to control for the practice effect and to use adequate statistical methods (e.g., the Reliable Change Index [159]). Similarly, Parsons [24] highlighted the need for correspondence, representativeness, expedience, and relevance in VR research. Future research should better control for social factors (e.g., interaction with avatars) in VR research on executive functions. Concerning VR training of executive functions, it is important to include follow-ups in the design of the research, in order to understand the long term effects of such training. It is important to adopt an interdisciplinary approach, with more comprehensive behavior analysis, for research on executive functions and rehabilitation of dysfunction thereof in schizophrenia patients. This should be based on VR tools with complex real-life situation scripts combined with physiological measurements (heart rate variability, galvanic skin response, and blood pressure) and brain activity measurement carried out with different methods [158]. Augmented reality, which allows the projection of items and avatars onto actual scenes, is another interesting new direction for research.

Some clinical implications need to be discussed. Firstly, for both diagnosis and therapy, it is important to find the right balance between maximizing patient benefit and avoiding harm. Secondly, due to the lack of standardized VR tools for executive function assessment in schizophrenia, which includes guidelines on development, norming, psychometric validation, and administration, they should be used in combination with traditional tools of proven psychometric value [26,142]. Similarly, due to the lack of empirically verified and standardized therapeutic methods, VR tools should be used in combination with traditional, empirically verified methods and cognitive-behavioral therapy. The American Academy of Clinical Neuropsychology and the National Academy of Neuropsychology advises that innovative computer diagnostic and therapeutic methods should always be precisely described to provide comprehensive information on the product and thus to allow clinicians to make informed decisions with patient welfare in mind [160].

## 10. Conclusions

Executive dysfunction in schizophrenia is an important clinical and social problem. Impairments in monitoring, attention shifting, planning, inhibition, rule generation, abstract thinking, and working memory, as well as the loss of skills required for the performance of complex tasks, can negatively impact patients’ social and professional functioning as well as their quality of life. When diagnosing executive dysfunction, an array of neuropsychological methods must be used—not only traditional tools, but also those with proven ecological validity (i.e., ecological and VR tools)—to accurately understand the underlying nature of the deficits observed. However, due to the many potential risks of using VR in clinical conditions and the lack of standardized VR tools for executive function assessment in schizophrenia, they should be used in combination with traditional tools of proven psychometric value. Future research should be based on paradigms that allow the use of VR assessment of executive functioning with methods of functional neuroimaging. Moreover, it would be interesting to investigate the influence of psychiatric medications on executive functioning measured by VR techniques in schizophrenia.

## Figures and Tables

**Table 1:** Characteristics of and evidence for diagnostic validity and two dimensions of ecological validity (verisimilitude and veridicality) of traditional, ecological, and VR tools that measure executive functions in schizophrenia.

Type	Tool	Diagnostic Validity		Ecological Validity				
				Verisimilitude			Veridicality	
		Examined	Confirmed	Developed	Construct	Technological Demands	Examined	Confirmed
Traditional	SCWT [85,86]	Yes	Yes: significant difference between patients with schizophrenia and healthy controls [1,87,88]; several meta-analyses	No	Laboratory task: paper-pencil or computerized task	Computer version as non-immersive technology	Yes	Yes: significant correlation between results on SCWT and scales of community functioning [89,90,91]; several meta-analyses
Traditional	TOL [85,92]	Yes	Yes: significant difference between patients with schizophrenia and healthy controls [93,94,95]; several meta-analyses	No	Laboratory task: paper-pencil or computerized task	Computer version as non-immersive technology	Yes	Yes: significant correlation between results on TOL and scales of community functioning, scales of social behavior in the milieu, scales of social problem-solving, scales of social skills [7]; one meta-analysis
Traditional	TMT [85]	Yes	Yes: significant difference between patients with schizophrenia and healthy controls [1,87,88]; several meta-analyses	No	Laboratory task: paper-pencil or computerized task	Computer version as non-immersive technology	Yes	Yes: significant correlation between results on TMT and scales of community functioning [89,91]; two meta-analyses
Traditional	WCST [85,96]	Yes	Yes: significant difference between patients with schizophrenia and healthy controls [1,2,87,88]; several meta-analyses	No	Laboratory task: paper-pencil or computerized task	Computer version as non-immersive technology	Yes	Yes: significant correlation between results on WCST and scales of community functioning, scales of social behavior in the milieu, scales of social problem-solving, scales of social skills [7]; one meta-analysis
Traditional	VFT [85,97]	Yes	Yes: significant difference between patients with schizophrenia and healthy controls [1,87,88]; several meta-analyses	No	Laboratory task: paper-pencil or computerized task	Lack of computer version	Yes	Yes: significant correlation between results on VFT and scales of community functioning [7]; one meta-analysis
Traditional	DS [85,98]	Yes	Yes: significant difference between patients with schizophrenia and healthy controls [1,87,99,100]; several meta-analyses	No	Laboratory task: verbal or computerized task	Computer version as non-immersive technology	Yes	Yes: significant correlation between results on DS and scales of community functioning [7,89,90,91]; several meta-analyses
Traditional	DST [101]		Yes: significant difference between patients with schizophrenia and healthy controls [10]; one meta-analysis	No	Laboratory task: verbal or computerized task	Lack of computer version	Yes	Yes: significant correlation between results on DST and scales of community functioning [7]; one meta-analysis
Traditional	LNST [102]	Yes	Yes: significant difference between patients with schizophrenia and healthy controls [1]; one meta-analysis	No	Laboratory task: verbal or computerized task	Computer version as non-immersive technology	Yes	Yes: significant correlation between results on LNST and scales of community functioning [91]; one meta-analysis
Traditional	SS [85,103]		Yes: significant difference between patients with schizophrenia and healthy controls [99,100]; two meta-analyses	No	Laboratory task: manual or computerized task	Computer version as non-immersive technology	Yes	Yes: significant correlation between results on SS and scales of community functioning [89,90,91]; several meta-analyses
Ecological	EFPT [104,105]	Yes	Yes: significant difference between patients with schizophrenia and healthy controls [104]; single study	Yes	Independent living as manual task: simple cooking, telephone use, medication management, and bill payment	Computer version as non-immersive technology	No	No information
Ecological	MET [106,107]	Yes	Yes: significant difference between patients with schizophrenia and healthy controls [106,108]; two studies	Yes	Manual task: shopping center	Computer version as non-immersive technology	Yes	Yes: significant correlation between results on MET and two scales of real-world functioning [106]; single study No: no significant correlation between results in MET and two scales of real-world functioning [108]; single study
Ecological	MSET [109]	Yes	Yes: significant difference between patients with schizophrenia and healthy controls [110]; one meta-analysis	Yes	Manual tasks: six realistic problems	Lack of computer version	Yes	Yes: significant correlation between MSET and scale of real-world functioning [111]; single study No: no significant correlation between results on MSET and questionnaire ratings of executive impairment [112]; single study
Ecological	ZMT [113,114]	Yes	Yes: significant difference between patients with schizophrenia and healthy controls [110]; one meta-analysis	Yes	Manual task: map of zoo	Lack of computer version	Yes	Yes: significant correlation between ZMT and questionnaire ratings of executive impairment and scale of real-world functioning [111,112]; two studies
Virtual reality	VAP-S [115]	Yes	Yes: significant difference between patients with schizophrenia and healthy controls [116]; single study	Yes	Virtual reality task: supermarket with aisles displaying items and static virtual people (avatars)	Computer non-immersive technology, scenario, participants’ activities in the environment, graphic representations of real items	Yes	Yes: significant correlation between results on VAP-S and results of naturalistic behavioral observations of real-life tasks [117]; single study
Virtual reality	VCFMT [118]	Yes	Yes: significant difference between patients with schizophrenia and healthy controls [118]; single study	Yes	Virtual reality task: city, station, bus and virtual people (avatars)	Computer non-immersive technology, scenario, participants’ activities in the environment, graphic representations of real items	No	No information
Virtual reality	CMPT ^a^ [119]	Yes	Yes: significant difference between patients with schizophrenia and healthy controls [119]; single study	Yes	Virtual reality task: office in a company and virtual people (avatars)	Computer non-immersive technology, scenario, participants’ activities in the environment, graphic representations of real items	Yes	Yes: significant correlation between results on CMPT and two scales of real-world functioning [119]; single study
Virtual reality	CMPT ^b^ [120]	Yes	Yes: significant difference between patients with schizophrenia and healthy controls [120]; single study	Yes	Virtual reality task: office in a company and virtual people (avatars)	Computer non-immersive technology, scenario, participants’ activities in the environment, graphic representations of real items	Yes	No: no significant between results on CMPT and two scales of real-world functioning [120]; single study
Virtual reality	CST [121]	Yes	Yes: significant difference between patients with schizophrenia and healthy controls [121]; single study	Yes	Virtual reality task: grocery and virtual people (avatars)	Computer non-immersive technology, scenario, participants’ activities in the environment, graphic representations of real items	Yes	Yes: significant correlation between results on CST and scale of real-world functioning [121]; single study
Virtual reality	PDT [122]	Yes	Yes: significant difference between patients with schizophrenia and healthy controls [123,124]; two studies	Yes	Virtual reality task: workplace environment	Computer non-immersive technology, scenario, participants’ activities in the environment, graphic representations of real items	Yes	Yes: significant correlation between results on PDT and scale of real-world functioning [123,124]; two studies
Virtual reality	VEP [125]	Yes	Yes: significant difference between patients with schizophrenia and healthy controls [125]; single study	Yes	Virtual reality task: rooms with three doors in each and corridors	Computer immersive technology, scenario, participants’ activities in the environment, graphic representations of real items	No	No information
Virtual reality	VMT [126]	Yes	Yes: significant difference between patients with schizophrenia and healthy controls [126]; single study	Yes	Virtual reality task: maze with walls and green grass	Computer immersive technology, scenario, participants’ activities in the environment	No	No information
Virtual reality	VRPMT [127,128]	Yes	Yes: significant difference between patients with schizophrenia and healthy controls [128]; single study	Yes	Virtual reality task: shopping center with aisles displaying items and static virtual people (avatars)	Computer non-immersive technology, scenario, participants’ activities in the environment, graphic representations of real items	No	No information
Virtual reality	VSST [129]	Yes	Yes: significant difference between patients with schizophrenia and healthy controls [129]; single study	Yes	Virtual reality task: supermarket with aisles displaying items and static virtual people (avatars)	Computer non-immersive technology, scenario, participants’ activities in the environment, graphic representations of real items	Yes	No: no significant correlation between results on VSST and scale of real-world functioning [129]

^a^ CMPT without interruptions or unexpected outcomes and prospective memory instructions; ^b^ CMPT with interruptions or unexpected outcomes and prospective memory instructions. CMPT = Computerized Meeting Preparation Task; CST = Computerized Shopping Task; DS = Digit Span; DST = Digit Sequencing Test; EFPT = Executive Function Performance Test; LNST = Letter Number Sequencing Test; MET = Multiple Errands Test; PDT = Plan-a-Day Test; SCWT = Stroop Color Word Test; SET = Six Elements Test; SS = Spatial Span; TMT = Trail Making Test; TOL = Tower of London; VAP-S = Virtual Action Plan—Supermarket; VCFMT = Virtual Cognitive Flexibility Measurement Task; VEP = Virtual Egyptian Pyramids; VFT = Verbal Fluency Test; VMT = Virtual Maze Task; VRPMT = Virtual-Reality Prospective Memory Test; VSST = Virtual Supermarket Shopping Task; WCST = Wisconsin Card Sorting Test; ZMT = Zoo Map Test.

**Table 2:** Components of executive dysfunction in schizophrenia [130,131].

Aspect of Executive Dysfunction	Definition
Monitoring deficits	Problems with the detection and resolution of conflict or detection and correction of errors.
Inhibition deficits	Diminished ability to withhold automatic reactions or associations that are inappropriate to the currently executed task.
Attention shifting deficits	Inability to flexibly transition between at least two processes or tasks.
Planning deficits	Reduced ability to set sequences of steps that lead to the achievement of a particular goal.
Rule generation deficits	Impaired formation of mental representations of rules concerning ways to solve problems in new situations.
Abstract thinking deficits	Limitations in ability to break away from the current situational context and to transition to a different aspect that may not be directly related to the current state.
Working memory deficits	Storing and manipulating verbal or non-verbal information in a short period of time in order to carry out a task.

**Table 3:** Description of traditional neuropsychological tools used to assess executive functions in schizophrenia.

Tool	Description	Components
Stroop Color Word Test (SCWT [85,86])	Traditionally three independent tasks constitute the SCWT: quickly identifying the names of colors in black ink; quickly identifying colors depicted as rectangles; and quickly identifying colored words printed in ink of an incongruent color. Manual and computer versions.	Working memory, verbal attention, cognitive inhibition
Tower of London (TOL [85,92])	Participants are shown a wooden tower with three pins (small, medium, large) and three balls (green, blue, red); from a predetermined initial state, they are asked to move the balls, one by one, to match a desired end position shown on a picture (12 configuations in total), using the minimum necessary moves (between 2 and 5); if the participant cannot finish or uses more moves, they are asked to start over. Manual and computer versions.	Planning, working memory, visual attention
Trail Making Test (TMT [85])	This task is split into two parts. In part A, the participant connects 25 irregularly arranged circles containing numbers from 1 to 25; part B requires alternation between circles arranged irregularly with numbers (1 to 13) and letters (A to L) to form a continuous line. Paper-and-pencil test.	Non-verbal attention, working memory, non-verbal cognitive flexibility
Wisconsin Card Sorting Test (WCST [85,96])	WCST requires two identical decks of cards (each containing 64 cards) and four reference cards; a participant must place subsequent cards according to their shape, color, and number using the researcher’s feedback. Manual and computer versions.	Working memory, switching, perseveration, problem-solving
Verbal Fluency Test (VFT [85,97])	A letter (phonemic) or a category (semantic) task is used. Participants are asked to produce as many unique words as possible within 60 s from each category in the semantic task or words starting with a given letter in the phonemic task.	Working memory, verbal flexibility, word production, verbal attention
Digit Span (DS [85,98])	Digit Span is a core subtest of the Wechsler Intelligence Scales. It consists of two parts: (a) Digits Forward characterized as a simple span test in which participants are asked to repeat a series of digits in a correct order, (b) Digits Backward that requires repeating digits in the reverse order of presentation.	Verbal sustained attention, storage and manipulation of information in verbal working memory
Digit Sequencing Test (DST [101])	Participants are asked to order series of numbers from lowest to highest. Number of correct responses and the longest sequence recalled correctly are recorded.	Storage and manipulation of information in non-verbal working memory
Letter Number Sequencing Test (LNST [102])	Participants are asked to sequence a random order of numbers and letters (numbers in ascending order followed by letters in alphabetical order.	Storage and manipulation of information in non-verbal working memory
Spatial Span (SS [85,103])	Task consists of two parts: (a) Spatial Forward which requires participants to point the blocks in a given order, (b) Spatial Backward which requires pointing the blocks in the reverse order.	Visual sustained attention, storage and manipulation of information in non-verbal working memory

**Table 4:** Descriptions of the ecological neuropsychological tools used for measuring executive functions in schizophrenia.

Tool	Description	Components
Executive Function Performance Test (EFPT [104,105])	Completing four tasks related to self-maintenance and independent living: simple cooking, using a telephone, managing medications, and paying bills.	Working memory, multitasking, planning, activities of daily living
Multiple Errands Test (MET [106,107])	This test measures everyday executive functioning with real-world activities (e.g., purchasing specific items, arriving at a destination, collecting and writing down information). The sum and type of errors (such as rule breaks and omissions) are registered.	Strategy allocation, planning, working memory
Modified Six Elements Test (MSET [109])	This test consists of six tasks: two sets of arithmetic problems two sets of pictures to be named, and two dictation tasks; the participant attempts to solve them according to set rules within 10 min. Tasks of the same type cannot be completed one after the other.	Working memory, strategy application, performance monitoring, planning
Zoo Map Test (ZMT [113,114])	Participants plan a route for a visit to the zoo according to rules provided (e.g., starting point, number of possible locations, using paths just once).	Working memory, planning, multitasking

**Table 5:** Descriptions of virtual reality tools for measuring executive functions in schizophrenia.

Tool	Description	Components
Virtual Action Plan—Supermarket (VAP-S [115])	This task emulates a medium size supermarket and the goal is to make purchases based on the list provided. Total distance, time, number of products, correct and incorrect actions, and pauses are recorded. Computer version.	Cognitive planning, working memory, sustained attention
Virtual Cognitive Flexibility Measurement Task (VCFMT [118])	The goal of this task is to get to a meeting place by bus; the participant is provided with a destination and recommended bus route. The participant can review a bus route map and choose one from a number of options (wrong, slow, pre-informed, or quick). Computer version.	Decision making, cognitive flexibility, prospective memory, working memory
Computerized Meeting Preparation Task (CMPT [119])	The task consists of two parts: learning and meeting preparation. The learning phase familiarizes the participant with the design of the task, which involves progressive learning (total time and number of incorrect actions are recorded). During the meeting preparation phase, the participant is asked to organize a meeting according to the provided rules. The task requires, inter alia, placing guests, bringing office equipment, and preparing drinks. Total time, number of times the instructions were checked, incorrectly placed/missed objects, respect for rules, planning score, and initiation are scored. Computer and real-life version.	Planning, multitasking, working memory
Computerized Shopping Task (CST [121])	This task consists of two parts. The learning phase aims to make the participant familiar with the functions and actions available. For the main shopping task, the participant is presented with a list of seven items belonging to distinct categories. Total time, correct items, intrusions, aisle redundancy, corrected errors, number of times in a non-relevant aisle, number of times a non-relevant aisle was visualized, number of times the shopping list was checked, time spent checking the shopping list, and checking the shopping cart are recorded. Computer version.	Planning, multitasking, organizing, prospective memory, working memory
Plan-a-Day Test (PDT [122])	This task emulates a workplace environment. The goal of the task is to schedule a list of daily work-related activities based on a given list of tasks. Total time, planning ratio, and number of solved problems without correction are recorded. Computer version.	Planning, organizing, prospective memory, working memory
Virtual Egyptian Pyramids (VEP [125])	A participant walks through a series of rooms and corridors in a pyramid. One of three doors needs to be chosen in each room. Doors are labeled with a shape, a color, and a sound. The task mechanics resemble the WCST. Computer version.	Switching, perseveration, problem-solving, working memory, integration of multimodal stimuli
Virtual Maze Task (VMT [126])	The maze consists of six identical two-way intersections. Corridors lead either to another intersection or to one of seven cul-de-sacs. Only one dead end contains money (the goal). Five trials with a 5 min time limit are given to reach the goal. Computer version.	Spatial learning, decision making, working memory
Virtual Reality Prospective Memory Test (VRPMT [127,128])	An adaptation of the Virtual Reality Shopping Task [127]. It imitates a shopping center with 20 stores. Obtaining items from a shopping list is used as a distraction task. Execution of time-based and event-based prospective memory tasks are recorded. Computer version.	Prospective memory
Virtual Supermarket Shopping Task (VSST [129])	Simulates shopping activity in a supermarket. Participants are asked to memorize and collect items from a list. A shopping list cannot be consulted and there is no time limit. Number of correct items, errors, trial time, and trial distance are scored. Computer version.	Planning, multitasking, working memory

## Data Availability

Materials of the review reported here are available from the corresponding author on reasonable request.
